# Eccentric Training Improves Body Composition by Inducing Mechanical and Metabolic Adaptations: A Promising Approach for Overweight and Obese Individuals

**DOI:** 10.3389/fphys.2018.01013

**Published:** 2018-08-07

**Authors:** Valérie Julian, David Thivel, Frédéric Costes, Julianne Touron, Yves Boirie, Bruno Pereira, Hélène Perrault, Martine Duclos, Ruddy Richard

**Affiliations:** ^1^Service de Médecine du Sport et Explorations Fonctionnelles, CHU Clermont-Ferrand, INRA, CRNH, Université Clermont Auvergne, Clermont-Ferrand, France; ^2^Laboratoire AME2P, Université Clermont Auvergne, Clermont-Ferrand, France; ^3^INRA, CRNH, Université Clermont Auvergne, Clermont-Ferrand, France; ^4^Service de Nutrition Clinique, CHU Clermont-Ferrand, INRA, CRNH, Université Clermont Auvergne, Clermont-Ferrand, France; ^5^Service de Biostatistique, CHU Clermont-Ferrand, Université Clermont Auvergne, Clermont-Ferrand, France; ^6^Faculté des Sciences de la Santé, Université d'Ottawa, Ottawa, ON, Canada

**Keywords:** eccentric exercise, concentric exercise, fat mass, lean mass, obesity, metabolism, training effects

## Abstract

Skeletal muscle generates force by either shortening (concentrically) or lengthening (eccentrically). Eccentric (ECC) exercise is characterized by a lower metabolic demand and requires less muscle activity than concentric (CON) exercise at the same level of exerted force. However, the specific effect of ECC training vs. CON training on lean and fat mass remains underexplored. The first aim of this paper was to review the available evidence regarding the effects of ECC training on whole body and segmental lean and fat mass and, when possible, compare these with the effects of CON training. The second aim was to provide some insights into the main mechanical, physiological, and metabolic adaptations of ECC training that contribute to its effects on body composition. The third aim was to determine the beneficial effects of ECC exercise on health-related parameters in overweight and obese patients. ECC training is an effective modality to improve lean mass, but when matched for load or work, the difference between ECC and CON trainings seems unclear. A few studies reported that ECC training is also efficient at reducing fat mass. By increasing post-exercise resting energy expenditure, modifying metabolic substrate, and improving both blood lipid profile and insulin resistance, ECC training is a potential exercise modality for individuals with chronic conditions such as those who are overweight and obese. Further investigations using standardized experimental conditions, examining not only segmental but also whole body composition, are required to compare ECC and CON trainings.

## Introduction

Daily life activities are performed with a combination of concentric (CON) and eccentric (ECC) muscle contractions. CON contractions serve to generate motor actions, whereas ECC contractions are involved in the braking movements of externally loaded muscles. ECC training involves isoinertial or isokinetic segmental contractions (Coratella et al., 2015). Recently, training alternative modalities, such as downhill walking or running, or cycling with special motorized eccentric cycle-ergometers (Rakobowchuk et al., 2018), have been conceived and characterized as “continuous moderate load eccentric exercise” (Hoppeler, 2016). Hoppeler (2016) characterized three types of ECC training into plyometric exercises (such as drop jumps, with contractions lasting milliseconds and producing thousands of watts of negative power), classical ECC resistance exercises (protocols consisting of near maximal ECC contractions lasting few seconds, used to lift and lower weights), and “continuous moderate load ECC exercises” [also as denoted (RENEW) Resistance Exercise via Negative Eccentric Work by LaStayo et al. (2017)]. For the same mechanical power, oxygen consumption (V°O_2_) during downhill running is approximately half compared with that during uphill running and is three to four times lower during ECC cycling than that during CON cycling (Perrey et al., 2001; Dufour et al., 2004). Continuous moderate load ECC exercise is characterized by lower metabolic and cardiorespiratory demands than CON exercise when performed at the same power output (Perrey et al., 2001; Minetti et al., 2002; Dufour et al., 2004; Chavanelle et al., 2014). Thus, an increasing interest has emerged with its use in patients with chronic diseases (Isner-Horobeti et al., 2013; Hoppeler, 2016) that are accompanied by cardiac, respiratory, or muscular impairments. This ECC modality has been increasingly prescribed and is proposed to patients with cardiac problems (Hortobágyi and DeVita, 2000; Meyer et al., 2003; Steiner et al., 2004; Zoll et al., 2006; Theodorou et al., 2013), respiratory disabilities (Meyer et al., 2003; Steiner et al., 2004; Rocha Vieira et al., 2011), sarcopenia (LaStayo et al., 2003), or neurological and musculoskeletal diseases (Engardt et al., 1995; Hawkins et al., 1999; Gür et al., 2002).

As improving body composition is a major target of training programs, identification of the exact effects of CON and ECC modalities on both lean and fat mass is necessary. However, the specific effects of ECC training on lean and fat mass remain underexplored compared with CON training. Several methodological barriers make it difficult to compare CON and ECC trainings, including (i) the difficulty of isolating ECC and CON actions during usual movements of daily life, and (ii) the lack of simple comparisons of ECC and CON exercises in standardized experimental conditions of similar power output (i.e., at the same mechanical power), similar oxygen consumption (i.e., at the same metabolic rate), and similar intensity and work volume.

This narrative review aimed to summarize the available evidence about the effects of ECC training on whole body lean and fat mass, and on segmental body composition (i.e., of the trained part), and compare this with the effects of CON training or, when not possible, with traditional training (which are mainly executed in practice). We reviewed studies carried out over the last two decades in which ECC was used as a training modality using ECC exercises mobilizing “segmental” or “large muscle mass.” For greater consistency, we prioritized, whenever possible, continuous moderate load ECC training over classical ECC resistance training and focused our research on whole body and lower limb composition. The second aim was to provide some insights into the main mechanical, physiological, and metabolic adaptations of ECC training that contribute to its potential effects on body composition. Finally, we determined whether ECC training is an innovative and promising approach for the management of overweight and obesity. This review is aimed at exercise scientists, health professionals, and trainers in the field of chronic disease and rehabilitation.

## Effects of eccentric training on body composition

Evaluation of body composition depends on the assessment model used to access both lean and fat mass. Advanced technologies, such as dual-energy X-ray absorptiometry (DXA), computed tomography (CT), and magnetic resonance imaging (MRI), are used by researchers to precisely quantify both whole body and segmental body composition at specific sites. Systematic differences have been reported between DXA and CT or MRI measures of lean mass. DXA overestimates the measure of lean mass, which is attributed to the significant non-fat component of adipose tissue (Levine et al., 2000). DXA is generally preferred when tissue mass is needed as a denominator for metabolic measurements, and CT or MRI are more appropriate when appendicular tissue composition is needed for relating it to muscle strength. Thus, if appendicular composition is measured for the purpose of relating it to muscle strength, CT is the most satisfactory method, whereas if tissue mass is needed as a denominator for metabolic measurements, DXA is preferred (Levine et al., 2000). Ultrasonography is also currently used to assess lean body mass (muscle thickness and muscle volume; Abe et al., 2015) and body fat (Wagner, 2013; Váczi et al., 2014). Several other measurement techniques are available but we focused on the aforementioned reference methods, which are the most sensitive, safe, and relevant in clinical practice (Lemos and Gallagher, 2017).

### Eccentric training and lean mass

Direct measurements of segmental body composition performed before and after ECC training generally show an improvement in lean mass and volume in healthy subjects of a wide age range (including elderly subjects), as well as in patients with chronic pathologies. These lean mass improvements are summarized in Table 1 and compared either to CON or traditional training.

**Table 1:** Studies analyzing the effects of eccentric training on lean mass.

**Studies**	**Population**	**Technique for body composition measurements**	**Training intervention**	**Training duration**	**Effects**
Blazevich et al., 2007	Active young adults Mean age: 22.5 years CON *n* = 12; ECC *n* = 12 (17 F; 16 M)	MRI (Whole quadriceps volume)	ECC: 4–6 sets of 6 reps (knee extension on an isokinetic dynamometer), 50–90%MVC. Volume (sets × reps × load): 1,200 to 3,240 CON: 4–6 sets of 6 reps (knee extension on an isokinetic dynamometer), 50–90%MVC. Volume (sets × reps × load): 1,200 to 3,240	10 weeks (3 x/week)	+10% *p* < 0.001 NS between groups
Marcus et al., 2008	Adults with type 2 diabetes mellitus Mean age: 50.7 years AE *n* = 8; AE/RE *n* = 7 (7 F; 8 M)	MRI (Thigh lean tissue CSA)	AE: aerobic exercise (treadmill, recumbent stepper, stationary bicycle, rowing machine). Intensity: 60–85% of age-predicted heart rate. Duration: 50 min. Workload not available AE/RE: recumbent eccentric stepper. Intensity: RPE from “very very light” to “somewhat hard”. Duration: 20 min + aerobic training to complete 50 min. Workload not available	16 weeks (3 x/week)	AE: – 4% *p* < 0.05 AE/RE: + 10.5% *p* < 0.05
Marcus et al., 2009	Post-menopausal women Mean age: 56.1 years CT *n* = 6; ECC *n* = 10	DXA (Leg lean mass)	ECC: eccentric ergometer. Intensity: RPE from “very very light” to “somewhat hard”. Duration 30 min. Work increased from 20.3–229.7 kJ. CT: control group with no supervised program	12 weeks (3 x/week)	ECC: + 6% *p* < 0.05 CT: – 0.03% NS
Mueller et al., 2009	Older adults Mean age: 80.6 years CT *n* = 14; RET *n* = 21; EET *n* = 19 (36 F; 26 M)	DXA (Thigh muscle mass)	EET: eccentric ergometer. Intensity: initially set at 30 W for women and 50 W for men, gradually ramped up according to RPE and DOMS. Duration 20 min. Workload not available RET: 3 sets of 8–10 reps (leg press, knee extension, leg curl, hip extension) with a progressive load based on RPE and DOMS. Duration 20 min. Load was increased if subject was able to do 10 reps. Intensity not described. Workload not available CT: computer-guided cognitive training without physical training	12 weeks (2 x/week)	EET: + 2.5% *p* < 0.05 RET: + 2% *p* < 0.05 CT: + 0.4% NS
LaStayo et al., 2009	Older and obese adults Mean age: 67.5 years ECC *n* = 9; TRAD *n* = 8 (13 F; 4 M)	MRI (Whole quadriceps volume)	ECC: eccentric stepper. Intensity ramped according to RPE from “fairly light” to “somewhat hard”. Duration 30 min. Mean work increased from 44 003 to 86 480 kJ TRAD: 3 sets of 10–12 reps (leg press, leg curl, leg extension, calf raise). Intensity 70% 1 RM. Relative volume 2,100–2,520	12 weeks (3 x/week)	ECC: + 11.5% *p* < 0.001 TRAD: + 3% NS
Reeves et al., 2009	Older adults Mean age: 70 years ECC *n* = 10; CONV *n* = 9 (10 F; 9 M)	Ultrasonography *(Vastus lateralis* thickness)	ECC: 2 sets of 10 reps (knee extension, leg press), intensity 80% 5 RM. Absolute volume (sets × reps × load) 157,525 ± 47,790 CONV: 2 sets of 10 reps (knee extension, leg press), intensity 80% 5 RM. Absolute volume (sets × reps × load) 197,722 ± 77,461	14 weeks (3 x/week)	ECC: + 11% *p* < 0.05 CONV: + 11% *p* < 0.05
Raj et al., 2012	Older adults Mean age: 68 years EB *n* = 13; CONV *n* = 12 CT *n* = 13 (11 F; 17 M)	Ultrasonography *(Vastus lateralis* thickness)	EB: 3 sets of 10 reps (leg press, toe press, pull-down, bench press), intensity 50% 1 RM. Relative volume (sets × reps × load) 1,500 CONV: 2 sets of 10 reps (leg press, toe press, pull-down, bench press), intensity 75% 1 RM. Relative volume (sets × reps × load) 1,500 CT: no exercise	16 weeks (3 x/week)	EB: + 5% *p* < 0.05 CONV: + 0% NS CT: – 6% *p* < 0.05
English et al., 2014	40 healthy males Mean age: 34.9 years CON *n* = 8; ECC 33 *n* = 8; ECC 66 *n* = 8; ECC 100 *n* = 8; ECC 138 *n* = 8	DXA (Whole body lean mass and leg lean mass)	CON: 2–5 sets of 2–8 reps (supine leg press and supine calf press). Intensity modified each session from 55–96% 1 RM ECC 33: program based on CON + ECC training (leg press) at 33% of the CON load ECC 66: program based on CON + ECC training (leg press) at 66% of the CON load ECC 100: program based on CON + ECC training (leg press) at 100% of the CON load ECC 133: program based on CON + ECC training (leg press) at 138% of the CON load	8 weeks (3 x/week)	Whole body lean mass NS in any group Leg lean mass ECC 133: + 2.4% *p* < 0.05 NS in other groups
Váczi et al., 2014	Active older males Mean age: 65 years ECC *n* = 38; SCC *n* = 9 (57 F; 20 M)	MRI (Quadriceps CSA)	ECC (knee extension on an isokinetic dynamometer): 4 sets of 8 to 14 reps. Intensity based on %MVC. SCC: Stretch load/contractions from 86 ± 24 J to 120 ± 10 J. Total work matched in the 2 groups.	10 weeks (3 x/week)	ECC: + 3% *p* < 0.05 SCC: + 2% *p* < 0.05
Franchi et al., 2015	Young males Mean age: 23 ± 4 *n* = 10 1 leg CON; 1 leg ECC	Ultrasonography (Quadriceps thickness) DXA (Thigh lean mass)	80% of CON or ECC 1 RM CON: 4 sets of 8–10 reps (leg press). Intensity 80% 1 RM CON. ECC: 4 sets of 8–10 reps (leg press). Intensity 80% 1 RM ECC. Relative loads matched.	4 weeks (3 x/week)	Muscle thickness ECC: + 7.5% *p* < 0.001; CON: + 8.4% *p* < 0.001 Thigh lean mass ECC: + 2.3% *p* < 0.01; CON: + 3% *p* < 0.01 NS between groups
LaStayo et al., 2017	Older adults fallers Mean age: 76.1 *n* = 134 (87 F; 47 M)	MRI (Thigh lean tissue CSA)	TRAD: circuit training, static tasks, aerobic exercise (cycle ergometer), flexibility exercises, upper extremity resistance exercise (free weights). 3 sets of 15 reps (leg press, standing multidirectional straight leg). Intensity 60–70% 1 RM. Duration 1 h. Workload not available. RENEW: circuit training + eccentric stepper. Intensity ramped according to RPE from “very very light” to “somewhat hard.” Duration 15 min. Total training duration 1 h. Workload not available	12 weeks (3 x/week)	Pre-post difference *p* < 0.05 in each group but NS between groups

*AE, aerobic exercise; AE/RE, aerobic exercise/resistance exercise; CON, concentric; CONV, conventional resistance training; CSA, cross-sectional area; CT, control group; DOMS, delayed onset muscular soreness; DXA, dual-X-ray absorptiometry; EB, Eccentrically biased resistance training; ECC, eccentric; EET, eccentric ergometer training; MRI, magnetic resonance imaging; MVC, maximal voluntary contraction; NS, non-significant; RET, conventional resistance training; RM, repetition maximum; RPE, rating of perceived exertion; SSC, stretch-shortening cycle; TRAD, traditional resistance training program; RENEW, resistance exercise via negative eccentric work*.

Several studies revealed significantly “greater effectiveness” of ECC in comparison with CON or traditional training. Raj et al. (2012) demonstrated that a 16-week ECC resistance training in older adults is superior to a conventional resistance training matched for the same relative volume. In older and moderate obese adults, another study showed that a 12-week moderate load ECC training (with ECC cycle-ergometers) exhibits a greater increase in quadriceps muscle size than a conventional resistance training (LaStayo et al., 2009). Similar results were obtained by Marcus et al. (2008) in a high-metabolic risk population of patients with type 2 diabetes: a 16-week program combining moderate load ECC exercises (with ECC cycle-ergometers) and aerobic exercises induces a superior increase in the thigh lean tissue cross-sectional area (CSA), compared with a program based on aerobic exercises only.

Other studies indicated similar results with regard to gain in lean mass after both forms of training. In elderly subjects with sarcopenia, Mueller et al. (2009) compared a 12-week moderate load ECC training group (with ECC cycle-ergometers) with a conventional resistance training group. They observed that both trainings induce a significant gain in thigh lean mass, without any significant difference between the groups. In older adult fallers, LaStayo et al. (2017) compared a moderate load ECC training group (the RENEW group, training with ECC cycle-ergometers) with a traditional resistance training group. Similarly, they found no significant difference in the increase in the CSA of thigh lean tissue after 12 weeks of training. In healthy active men and women, a 10-week ECC or CON resistance training matched for relative volume shows similar improvements in whole quadriceps volume (Blazevich et al., 2007). In young males, Franchi et al. (2015) also compared an ECC training with a CON resistance training matched for relative loads and found that quadriceps thickness and thigh lean mass increase with similar proportions after 4 weeks. In older adults, Reeves et al. (2009) also demonstrated similar improvements in *vastus lateralis* thickness after 14 weeks of resistance training (however, the volume of the ECC program was lower than that of the conventional program). Interestingly, English et al. (2014) demonstrated that an 8-week resistance ECC training in healthy males produces a superior increase in leg lean mass compared with CON training, provided that ECC loads were superior to CON.

Thus, both forms of exercise are effective to induce gains in lean mass. The studies reported above indicate that ECC training is more effective (LaStayo et al., 2009; Marcus et al., 2009; Raj et al., 2012; English et al., 2014) or at least as effective as other exercise modalities (Blazevich et al., 2007; Mueller et al., 2009; Reeves et al., 2009; Franchi et al., 2015) in improving segmental lean mass. Nevertheless, the greater effectiveness of ECC training in improving lean mass is not clearly elucidated in recent reviews. Franchi et al. (2017) discussed the contribution of ECC and CON resistance trainings to muscular hypertrophy. They grouped studies in different categories depending on the index used to assess hypertrophy (and thus on the model of assessment) and concluded that the changes between ECC and CON trainings are similar when matched for load or work. Furthermore, Schoenfeld et al. (2017) conducted a systematic review comparing low- and high-load resistance training protocols and found similar hypertrophy between conditions.

The increase in muscle fiber volume induced by ECC training is also microscopically confirmed by measuring the CSA of the muscle fiber. LaStayo et al. (2000) compared 8-week trainings based on either ECC or CON cycling matched for metabolic rate (quantified as a percentage of the patients' peak heart rate) in healthy males. The ECC program show an increase of more than 50% in the CSA of the muscle fibers measured using biopsy from the middle part of the vastus lateralis, whereas CON training exhibited no changes (LaStayo et al., 2000). However, measurement of the CSA of the whole muscle was not performed. This increase in the CSA of the muscle fiber after ECC training has been validated by several studies (Hortobágyi et al., 1996; LaStayo et al., 2003; Vikne et al., 2006). Other studies showed similar positive changes in the size of the muscle fibers after ECC training compared with CON training (Jones et al., 1989; Meyer et al., 2003). Only few studies failed to detect any significant change after ECC training (Colliander and Tesch, 1990; Fisher et al., 2016).

Moreover, muscle strength measurement, muscle CSA measurement, and DXA evaluation of thigh lean mass are closely correlated (Levine et al., 2000). Hence, muscle strength measurement is considered as a reliable indirect measure of lean mass, and has been extensively studied. The findings have revealed that ECC training induces a significantly greater increase in appendicular total strength (i.e., the sum of CON, isometric and ECC peak torque) and ECC strength, whereas the difference in isometric and CON measures seems less significant (Roig et al., 2009). Thus, the increase in ECC strength after ECC training is greater than the gain in CON strength after CON training (Higbie et al., 1996; Vikne et al., 2006).

In summary, ECC training is effective at inducing gains in lean mass. Comparison between ECC and CON training programs in terms of mechanical power, metabolic rate, intensity, or volume remains methodologically difficult. Although ECC training was previously associated with greater muscle hypertrophy, the findings were extremely varied to clearly affirm its superiority over CON or traditional training. However, considering the lower metabolic demand of ECC exercise, it would be more efficient than CON training given the ratio of energy expenditure to net force or work production. Investigations comparing not only segmental but also whole body lean mass after ECC and CON trainings are necessary.

### Eccentric training and fat mass

The effects of ECC training on body fat mass remain underexplored. The available clinical evidences are detailed and summarized in Table 2. As no study has considered the variations of whole body or segmental fat mass in comparing ECC and CON training, we presented here indirect comparisons between ECC and traditional trainings. We noted a 12-week randomized controlled trial among elderly patients with sarcopenia that measured both thighs and whole body fat mass in three groups of patients: (i) the moderate load ECC training group (training with motorized eccentric ergometer with a power output based on the patients' perceived exertion); (ii) the conventional resistance training group; and (iii) the cognitive training group. Although the trainings were not matched for load, only the ECC ergometer training group showed a significant reduction in thigh fat content and whole body fat mass (Mueller et al., 2009). In a high-metabolic risk population of patients with type 2 diabetes mellitus, a 16-week intervention combining moderate load ECC exercises (on motorized cycle-ergometers) and aerobic exercises was compared against a program with aerobic exercises only. The two groups experienced a decrease in thigh intramuscular fat area, without any significant difference between the groups concerning intramuscular fat. Interestingly, a greater reduction in body mass index was observed in the ECC group (Marcus et al., 2008). Another study showed that after 12 weeks of multimodal exercise intervention in elderly patients with comorbidities and a history of falling, no significant difference in intermuscular adipose tissue was found after either the ECC program (paired with moderate load ECC exercises on motorized stepper ergometers) or the traditional training program (paired with traditional resistance training; Jacobs et al., 2014). However, in these studies, the total training loads were not available. Another study that enrolled post-menopausal women with impaired glucose tolerance evaluated the effect of a 12-week moderate load ECC training program (with motorized eccentric ergometers) and measured a significant increase in leg lean mass combined with a decrease in abdominal fat mass (total body fat was not reported). Although the study also enrolled a control group (a computer-guided cognitive training group), it lacked a CON group for comparison (Marcus et al., 2009).

**Table 2:** Studies analyzing the effects of eccentric training on fat mass.

**Study, year**	**Population**	**Technique for body composition measurements**	**Training intervention**	**Training duration**	**Effects**
Marcus et al., 2008	Adults with type 2 diabetes mellitus Mean age: 50.7 years AE *n* = 8; AE/RE *n* = 7 (7 F; 8 M)	MRI (Thigh intramuscular fat CSA)	AE: aerobic exercise (treadmill, recumbent stepper, stationary bicycle, rowing machine). Intensity: 60%−85% of age-predicted heart rate. Duration: 50 min. Workload not available AE/RE: recumbent eccentric stepper. Intensity: RPE from “very very light” to “somewhat hard”. Duration: 20 min + aerobic training to complete 50 min. Workload not available	16 weeks (3 x/week)	Thigh intramuscular fat CSA AE: – 2.2 cm^2^ *p* < 0.05 AE/RE: – 1.2 cm^2^ *p* < 0.05 NS between groups
Marcus et al., 2009	Post-menopausal women Mean age: 56.1 years CT *n* = 6; ECC *n* = 10	DXA (Abdominal fat mass)	CT: control group with no supervised program ECC: eccentric ergometer. Intensity: RPE from “very very light” to “somewhat hard”. Duration: 30 min. Work increased from 20.3 to 229.7 kJ	12 weeks (3 x/week)	Abdominal fat mass CT: + 2.5% NS; ECC: – 3.7% NS
Mueller et al., 2009	Older adults Mean age: 80.6 years CT *n* = 14; RET *n* = 21; EET *n* = 19 (36 F; 26 M)	DXA (Total body and thigh fat mass)	RET: 3 sets of 10 reps (leg press, knee extension, leg curl, hip extension) with a progressive load based on RPE and DOMS. Duration 20 min. Volume not available. EET: eccentric ergometer. Intensity: initially set at 30 W for women and 50 W for men, gradually ramped up according to RPE and DOMS. Duration: 20 min. Workload not available CT: computer-guided cognitive training without physical training	12 weeks (2 x/week)	Whole body fat mass RET: – 0.6% NS EET: – 5.0% *p* < 0.01; CT: + 1.4% NS Thigh fat mass RET: – 2.7% NS; EET: – 6.9% *p* < 0.01; CT: + 0.6% NS
Jacobs et al., 2014	Older adults Mean age: 75.5 years TRAD *n* = 38; ECC *n* = 9 (57 F; 20 M)	MRI (Thigh intermuscular adipose tissue)	TRAD: 3 sets of 15 reps (bilateral leg press), 3 sets of 15 reps (standing multidirectional straight leg exercise). Intensity 60–70% 1 RM + aerobic exercise. Duration: 60 min. Volume (sets × reps × load) 2 700–3 150 ECC: eccentric ergometer. Intensity: RPE from “very very light” to “somewhat hard.” Duration: 15 min + aerobic exercise to complete 60 min. Workload not available	12 weeks (3 x/week)	Thigh intermuscular adipose tissue TRAD: – 0.1% NS; ECC: – 0.1% NS

*AE, aerobic exercise; AE/RE, aerobic exercise/resistance exercise; CSA, cross-sectional area; CT, control group; DXA, dual-X-ray absorptiometry; DOMS, delayed onset muscular soreness; ECC, eccentric; EET, eccentric ergometer training; MRI, magnetic resonance imaging; NS, non-significant; RET, conventional resistance training; RM, repetition maximum; RPE, rating of perceived exertion; TRAD, traditional resistance training program*.

In summary, ECC training is effective at reducing fat mass. Studies comparing ECC vs. CON training have not been conducted. Nevertheless, referring to the previous studies that compared ECC training and traditional trainings, ECC training is more (Mueller et al., 2009) or at least as effective as traditional training (Marcus et al., 2008; Jacobs et al., 2014) in reducing fat mass. ECC training may simultaneously increase lean mass and decrease fat mass, which would contribute to preventing sarcopenia and a number of other age-related metabolic impairments (Marcus et al., 2008, 2009; Mueller et al., 2009). Nevertheless, studies on the effects of ECC training on fat mass are scarce and further investigations are required. In particular, studies that evaluate both segmental and whole body composition and that employ the same mechanical power, the same metabolic intensity, or the same total training load or volume when calibrating training interventions are necessary.

## Physiologic and metabolic effects of eccentric exercise

**Figure 1:**
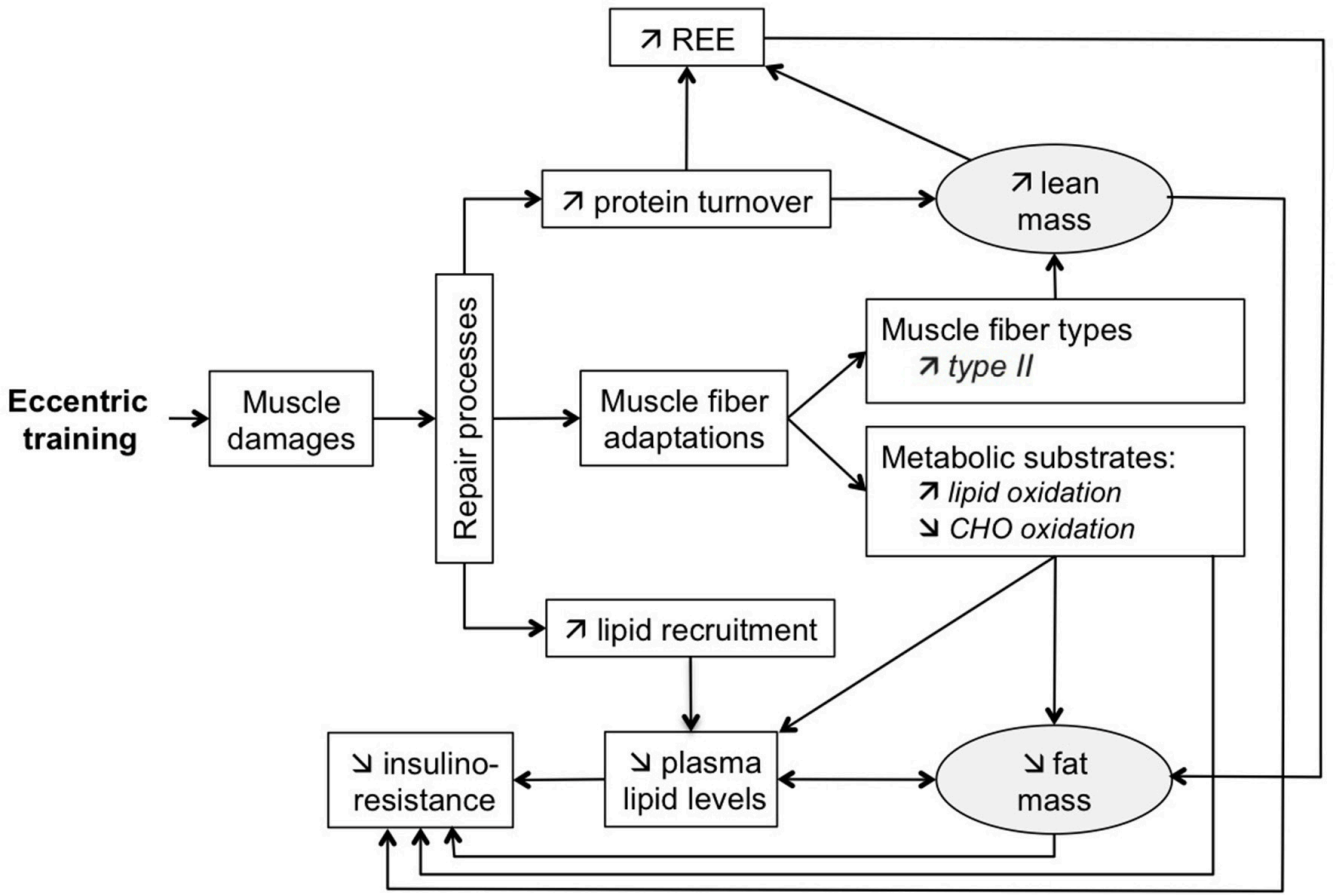
Schematic representation of physiological and metabolic effects of ECC training and their relationship with body composition changes.

Figure 1 schematically illustrates the physiological and metabolic effects of ECC training, and their relationship to changes in lean and fat mass.

### Eccentric training and resting energy expenditure

Resting energy expenditure (REE), which represents 60 to 75% of the total daily energy expenditure in humans, can remain elevated up to 48 h after a single bout of exercise (Melby et al., 1993; Gillette et al., 1994). Several studies have recently shown that ECC exercise may increase and prolong this rise in post-exercise REE more than CON exercise. A study conducted among healthy young women using oxygen calorimetry recorded a significant increase of 12% in REE 48 h after an acute segmental ECC exercise (five sets of 15 maximal voluntary contraction knee extensions), but not after a CON exercise set at the same power output (Paschalis et al., 2011). Some authors have even emphasized that acute ECC exercise can prolong the increase in post-exercise REE to at least 72 h (Hackney et al., 2008). This higher REE observed after an acute bout of ECC exercise may be caused by enhanced muscle protein turnover (i.e., an increase in both muscle protein degradation, which activates proteases and hydrolases, and muscle protein synthesis). The elevated turnover arises because of inflammatory processes to repair muscular myofibrillar damage and support muscle hypertrophy (Chesley et al., 1992; Burleson et al., 1998; Tidball, 2011). Although every exercise modality can lead to exercise-induced muscle damage (EIMD), ECC exercise has more damaging effects due to the higher force developed and the mechanical disruption of the actin-myosin bonds (Eliasson et al., 2006). EIMD is the result of both “mechanical” and “metabolic” stresses on the muscle fibers (Proske and Morgan, 2001; Allen et al., 2005; Tee et al., 2007), which cause temporary lesions of the cytoskeleton and disruption of the sarcomeres and Z-lines in response to overstretching (Proske and Morgan, 2001; Morgan et al., 2004; Tee et al., 2007). The unregulated influx of extracellular ions into the injured muscle induces elevation in cytosolic calcium concentration. This elevation leads to the activation of calcium-dependent proteases within the muscle cytosol (calpains), the activation of phospholipases and the elevation of free radical production, contributing to muscle damages (Tidball, 2011). These damages can be specific to a few macromolecules of muscular tissue or can involve large tears of sarcolemma, basal lamina and connective tissue (Vierck et al., 2000), with more damages to type II fibers (Douglas et al., 2017a). The increase in REE after acute ECC exercise is higher in untrained subjects than in trained subjects, suggesting that the degree of muscle damage is a strong determinant and that the repeated bout effect is increased in untrained subjects (Hackney et al., 2008). The inflammatory adaptations, the repair process mechanisms and the protein synthesis following ECC exercise are speculated to be limited after subsequent ECC bouts (McHugh, 2003; Lehti et al., 2007). It has been described that a bout of ECC exercise protects against muscle damages from subsequent ECC bouts (McHugh, 2003; Gault and Willems, 2013). Several mechanisms interact in this protective adaptation called the repeated bout effect, including neural adaptations with change in motor unit synchronization, alterations to muscle mechanical properties, structural remodeling of the extracellular matrix and inflammatory response (Hyldahl et al., 2017). The severity of EIMD increases with the intensity and duration of exercise (Lieber and Friden, 2002; Cheung et al., 2003), the untrained status of the subjects, aging, and chronic disease status (Tee et al., 2007; Tidball, 2011; Schoenfeld, 2012; Gault and Willems, 2013; Baumert et al., 2016). Other factors, such as the muscle involved (Jamurtas et al., 2005), muscle length (Paschalis et al., 2005), and angular velocity (Chapman et al., 2008) at which the exercise is performed, affect the extent and the duration of the EIMD.

A significant increase in REE is also observed in healthy young women after chronic ECC exercise compared with chronic CON exercise performed at the same power output (Paschalis et al., 2011). This increase may be attributed to the continuous adaptation of skeletal muscles to repair and regenerate after bouts of exercise and the larger increase in lean mass (described above) after ECC training. The inflammatory response to ECC exercise leads to increased content, recruitment and migration of satellite cells, thereby inducing muscle hypertrophy (Tidball, 2011; Peake et al., 2016). Once activated by the ECC mechanical stimulus, satellite cells generate myoblasts that proliferate and fuse to existing cells, increasing the number of myonuclei per cell (Tidball, 2011; Schoenfeld, 2012). Muscle repair is associated with novel transcriptional programs involving gene regulation, growth, and membrane synthesis (Schoenfeld, 2012). Moreover, mRNA production in the new myonuclear domains provides new muscle tissue (Petrella et al., 2008; Schoenfeld, 2012). *In fine*, the increase in the CSA of existing muscle fibers in response to ECC exercise may be associated with this expansion in myofibrillar content as a result of the activation of satellite cells, stimulation of anabolic signaling pathways, up-regulation of genes involved in anabolic mechanisms, and increase in protein translation and synthesis (Douglas et al., 2017b). As EIMD repair and regeneration processes decrease with training, the increase in post-exercise REE after chronic ECC exercises may occur due to both the increase in muscle protein turnover and gain in lean mass.

### Eccentric training, muscle fibers, and metabolic changes

Paschalis et al. (2011) showed that acute ECC exercise in untrained subjects can significantly modify metabolic substrate use, increasing post-exercise fat oxidation by 13% and reducing glucose oxidation. However, they observed no significant modification in metabolic substrates after exercise in the CON group at the same power output. This modification of substrate oxidation rates in response to ECC exercise might be due to a specific effect of ECC exercise on muscle metabolism. Considering muscle type fiber composition, greater increase in type II compared with type I muscle fibers has been extensively described in human subjects after ECC training (Hortobágyi and DeVita, 2000; Paddon-Jones et al., 2001; Friedmann-Bette et al., 2010; Douglas et al., 2017a), with a shift from IIx to IIa. According to Hody et al. (2013), the proportion of type I and IIa fibers significantly increases compared with type IIx or IIb fibers. By using a proteomic analysis approach without any pre-established hypothesis on humans trained with five sessions of ECC exercises, they observed a decrease in several glycolytic enzymes coupled with a lower expression of the fast isoforms of some contractile and structural proteins, suggesting that ECC training could result in a switch to more oxidative metabolism (Hody et al., 2011). A similar proteomic approach, completed with histological analyses of whole quadriceps muscles, was conducted in mice, comparing two groups submitted to five sessions of either uphill or downhill running (exercises performed at the same power output; Hody et al., 2013). The proteomic profiling confirmed the results previously observed in humans, showing that the ECC group had a lower abundance of the myosin heavy chain isoforms specific to fast-twitch glycolytic fibers compared with the CON group. When they combined the quantification of muscle fiber types and the calculation of the CSA of each muscle fiber type (i.e., the number of type I, IIa, and IIb fibers per square millimeter multiplied by the mean area), both the ECC and CON groups had a significantly higher relative surface area for slow oxidative fibers and a significantly lower surface area for fast glycolytic fibers than the untrained control group (Hody et al., 2013), and the ECC group had a significantly higher surface area for type I and IIa fibers than the CON group (Hody et al., 2013). The hypothesis of a higher oxidative muscle phenotype is also supported by blood profile analyses. In untrained subjects, both acute and chronic ECC exercises favor decreased triglyceride (TG), LDL cholesterol, and total cholesterol levels, along with improved HDL cholesterol levels (which were not significantly modified after similar CON exercises; Paschalis et al., 2011). This improvement in blood lipid profile may be associated with the increased demand of the working muscles for fatty acid substrates (coming from blood TGs and LDL cholesterol) to regenerate injured muscles and particularly to synthesize new cell membranes after ECC exercises (Drexel et al., 2008). Fatty acids are necessary for the replenishment of muscle phospholipids and TG stores for the regeneration of damaged muscle fibers (Nikolaidis et al., 2008). The observed increase in HDL cholesterol may be due to the heightened activity of the lipoprotein lipase after ECC exercise, which causes lipoprotein particles to shrink and transfer to HDL cholesterol (Frayn, 2003). TG plasma concentrations are also reduced by this rise in lipoprotein lipase, which acts on lipoprotein particles passing through the capillaries and releases free fatty acids. These may be taken up by muscles and then esterified in phospholipids and intramuscular TGs, or oxidized in the mitochondria (Nikolaidis et al., 2008). Thus, the improvement in lipid and lipoprotein profiles may be aided by the increase in lipid oxidation rate previously described after acute and chronic ECC exercise (Paschalis et al., 2011). The improvement in lipid profiles after ECC training may be attenuated in trained patients, partially because of a lower muscle repair activity owing to diminished EIMD (Nikolaidis et al., 2008).

With regard to insulin resistance, several studies have reported that an acute ECC exercise increases glycemia, insulin levels, and homeostasis model assessment of insulin resistance (Tee et al., 2007; Paschalis et al., 2011). This transitory increase in insulin resistance may be caused by EIMD, which may impair insulin signal transduction via inflammatory processes and decrease glucose transporter proteins (Tee et al., 2007). Nevertheless, most studies (Drexel et al., 2008; LaStayo et al., 2008; Paschalis et al., 2011; Zeppetzauer et al., 2013), but not all (Marcus et al., 2009; Philippe et al., 2016), demonstrated that the adverse effects of acute ECC exercise subside after chronic ECC exercise and that ECC training would decrease insulin resistance. An 8-week ECC training program proved sufficient to increase insulin sensitivity and improve lipid profile (Paschalis et al., 2011). The improvement in insulin sensitivity after ECC exercise may result from improved mitochondrial function and increased fat oxidation, which prevent the accumulation of fatty acid-derived metabolites in skeletal muscle (such as long chain acyl-CoA, diacylglycerol, and ceramides, which can in turn impair insulin signal transduction) (Rigalleau et al., 1998; Christ-Roberts et al., 2004; Houmard, 2007). The associated decrease in plasma TG may also be involved in the decrease in insulin resistance, as it can impair insulin action through over-activity of the glucose-fatty acid cycle (Randle et al., 1963). Moreover, these improvements in blood lipid profile and insulin resistance are combined with a decrease in low-grade inflammation, as proved by Drexel et al. (2008) in a chronic downhill hiking model of ECC training and confirmed by other studies (LaStayo et al., 2008; Zeppetzauer et al., 2013). Low-grade inflammation contributes to impaired insulin signal transduction, and reduction of such inflammation contributes to improving insulin sensitivity (Barrett and Eringa, 2012). Moreover, both lowering fat mass and increasing fat free mass are determinant factors that strongly influence insulin resistance (Macor et al., 1997; Kelly, 2000; Fernández-Real et al., 2003; Srikanthan and Karlamangla, 2011; Alemán-Mateo et al., 2014).

## Eccentric training for overweight and obese patients

Obesity is a major public health challenge. It is defined as an excessive fat accumulation due to an imbalance between energy intake and energy expenditure (Ebbeling et al., 2002). Obesity is associated with numerous comorbidities, such as type 2 diabetes, dyslipidemia, high blood pressure, cardiovascular disease, respiratory disease, and joint disease (Daniels et al., 2005). Educational strategies, in particular the combination of nutritional and physical interventions, are required to counteract the progressive metabolic disorders and functional impairments associated with obesity (Machado et al., 2016). Physical activity is effective for both preventing and controlling obesity, hypertension, type 2 diabetes, metabolic syndrome and cardiovascular diseases, because it contributes to better blood glucose control, reduced weight and waist circumference, and improved body composition, with benefits to the cardiovascular system (Machado et al., 2016). Nevertheless, patients with overweight and obesity present muscular, respiratory, or cardiac limitations with regard to their exercise capacities (Suastika, 2006; Look AHEAD Research Group and Wing, 2010). These limitations frequently reduce the intensity and duration of training exercises. Multidisciplinary programs are often used and are mainly based on CON activities, mostly due to limited access to ECC ergometers (because of the bespoken design and financial constraints). ECC exercise produces less cardiovascular and respiratory stress (Meyer et al., 2003) and less fatigue (Horstmann et al., 2001) than CON exercise, and requires lower metabolic demand than equal CON exercise (Perrey et al., 2001; Minetti et al., 2002; Dufour et al., 2004; Chavanelle et al., 2014). Although the energy expenditure during ECC exercise is lower than that during CON exercise when power output is matched, ECC exercise should be considered more efficient compared with CON exercise in increasing post-exercise REE (Paschalis et al., 2013) and thus in improving energy balance and weight management over time. Moreover, moderate load ECC training could be a strategy to maximize compliance, as it induces effective stimulations that not exceed the cardiorespiratory capacity of a patient (Mitchell et al., 2017). Moderate load ECC training is well-tolerated in multiple chronic conditions and critically ill patients (LaStayo et al., 2014; Mitchell et al., 2017).

The modality of muscle actions has not been completely studied in all aspects of energy balance, particularly in food intake. In the study by Paschalis et al. (2011) conducted in healthy subjects, daily energy and macronutrient intakes do not significantly change after ECC and CON training. Brain-derived neurotrophic factor (BDNF), which increases its production after muscle contraction in rodent and humans, is a growth factor that induces neurogenesis, protects against neurodegeneration, positively influences neural plasticity (learning and memory), plays a central role in fuel metabolism (fat oxidation), and is a strong central regulator of energy intake (Hu and Russek, 2008; Matthews et al., 2009; Yarrow et al., 2010). Several investigations in humans and rodents have supported the role of BDNF in regulating energy balance. In mice, global BDNF haploinsufficiency or brain-specific BDNF depletion results in excessive feeding and body weight gain accompanied by other features of the associated metabolic syndrome, including hyperleptinemia, hyperglycemia, and hyperinsulinemia (Rios et al., 2001; Beckers et al., 2008; Skledar et al., 2012). The BDNF levels in mice increase BDNF significantly (more than double) after ECC running compared with CON running (Aguiar et al., 2008). Moreover, BDNF increases in both the hippocampus and striatum after ECC running, but it increases in the hippocampus only after CON running (ECC and CON exercises were performed at the same mechanical power). This finding indicates that BDNF levels are most responsive after ECC exercise than CON exercise (Aguiar et al., 2008). In human subjects, Yarrow et al. (2010) demonstrated that an eccentric-enhanced progressive resistance training intervention enhances the transient post-exercise elevation of circulating BDNF. Conclusive research may be necessary to compare food intakes and biological regulators of appetite after acute and chronic ECC compared with CON exercise.

Overweight and obese patients are more sedentary than lean subjects, which in turn keep them in positive energy balance. They are also less active with regard to ECC exercise (Pacy et al., 1986). Biopsies obtained from vastus lateralis in patients with obesity show a higher proportion of type IIb muscle fibers (Kriketos et al., 1997), which are preferentially recruited and damaged during and after ECC exercise (Nardone and Schieppati, 1988). Since these patients are untrained, they exhibit higher EIMD and display more significant and more prolonged muscle alterations after ECC exercise than lean patients (Paschalis et al., 2013). Concerning post-exercise REE, Paschalis et al. (2011) demonstrated that patients with overweight and obesity exhibit significantly larger (+25% in overweight and obese subjects *vs*. +9% in lean subjects) and more prolonged (up to 72 h) increases in REE after acute ECC exercises, even in relation to their fat free mass. Additionally, their results showed that acute ECC exercise induces a significantly greater increase in lipid oxidation in overweight and obese patients than in lean subjects (Paschalis et al., 2011). This is particularly relevant because of the following three reasons. First, patients with obesity usually exhibit a lower rate of lipolysis (Blaak et al., 1994). Second, body fat content significantly positively correlates with type IIx fibers (Blaak et al., 1994). Third, oxidative enzyme activities negatively correlate with insulin resistance (Kriketos et al., 1996). This greater increase in lipid oxidation may arise from the increase in the hydrolysis of fatty acid phospholipids of the damaged muscle membranes and the alteration of the glucose transport system and insulin resistance following ECC exercise (King et al., 1993; Asp and Richter, 1996). Furthermore, the magnitude of the response of circulating lipids in patients with obesity after an acute ECC exercise is likewise significantly higher than that in non-obese patients. This result confirms that the extent of muscle damage is a strong determinant of the improvements in post-exercise blood profile (Paschalis et al., 2010). Nikolaidis et al. (2008) examined the effect of a repeated muscle damage exercise on the time-course changes in blood lipids and lipoprotein profiles. They showed that the effect of a repeated session of ECC exercise induces a relatively less improvement compared with the first exercise. Nevertheless, the benefic effects of chronic ECC training on healthy subjects suggest that the decrease in circulating lipids may continue after bouts of ECC exercises (Paschalis et al., 2011). With regard to glucose metabolism and insulin resistance, ECC training similarly offers benefits to patients with metabolic risk factors (Drexel et al., 2008; Marcus et al., 2008; Miles et al., 2016). Thus, decreased insulin resistance, intra-myocellular lipid pool changes, and decreases in glucose oxidation after ECC exercises support the growing interest for the ECC modality for the management of metabolic diseases (Hughes et al., 2010; Beaven et al., 2014; Gavin et al., 2015). Notably, ECC exercise may protect against low-grade inflammation usually observed in patients with obesity (Nascimento et al., 2016).

## Limitations of eccentric exercise

For chronic patients, which exhibit severe muscle wasting (in relation to general deconditioning, nutrition, systemic inflammation and/or medication), dyspnea is often experienced during classical CON exercises. Moreover, skeletal muscle dysfunction limits patient tolerance to classical ECC exercises at relatively high load (close to maximal), which would make an evident choice for moderate load ECC exercises (Hoppeler, 2016). To date, a wide range of techniques and equipment, such as upper and lower limb ergometers (Isner-Horobeti et al., 2013; LaStayo et al., 2017), treadmills for walking downhill, and ergometers offering stair descending (Theodorou et al., 2013), facilitate ECC exercises. To reach a moderate training load, the supervision must be strict and require a specific experience. However, these moderate loads could be achieved without undue delayed onset muscular soreness (DOMS) provided that the initial 2- or 3-week progressive ramping protocol is followed (LaStayo et al., 2017). Nevertheless, the equipment is often bespoken and sophisticated, with financial constraints, which may also limit their expansion. These factors may contribute to the lack of studies comparing moderate load ECC training and other modalities of training. For specific cases of obese individuals, who display significant and prolonged muscle alterations after ECC exercise (Paschalis et al., 2013), the training may require an additional caution from the supervisor to adapt the progressive initial ramped phase to limit DOMS and to maximize exercise tolerance and training compliance. Nevertheless, the beneficial effect of ECC training on body composition and other health-related parameters make it a promising tool for this population.

## Conclusion

ECC training results in multi-target beneficial effects on lean and fat mass. Further investigations employing similar mechanical power, metabolic rate, intensity, and work volume when calibrating the ECC and CON exercises are required to provide a conclusive comparison of the two modalities. Our review focused on the effect of ECC training on lean and fat mass and particularly on whole body measurements, but more studies with better power and design are warranted. The heterogeneity of the available studies (in terms of populations, measurement techniques, etc.) makes it premature to draw any definitive conclusions (and explains why a meta-analysis was not possible here). EIMD after ECC exercise leads to local inflammation and regeneration, which enhance protein degradation and synthesis via activation of well-known intracellular hypertrophy signaling pathways. Both acute and chronic ECC exercises induce larger increases in post-exercise REE than acute and chronic CON exercises performed at the same power output, partially because of the increase in muscle protein turnover and gain in lean mass. Both acute and chronic ECC exercises can also modify metabolic substrate use by increasing post-exercise fat oxidation and reducing glucose oxidation, leading to a switch to a more oxidative metabolism. In line with the increased demand of the working muscle for fatty acid substrates to regenerate injured muscles, both acute and chronic ECC exercises improve blood lipid profile to a greater extent than CON exercises. Although a transient increase in insulin resistance occurs after acute ECC exercise because of EIMD, chronic ECC exercise also decreases insulin resistance.

ECC training, particularly continuous moderate load ECC training, is a potential exercise modality for overweight and obese patients because most metabolic and biological effects induced by ECC exercise are heightened in these subjects in comparison with lean subjects. Moreover, ECC exercise requires lower metabolic demands than CON exercises when performed at the same mechanical power and induces a greater increase in post-exercise REE. Further investigations using standardized experimental conditions in the ECC and CON training groups are necessary to define the specific metabolic effects of ECC training, to determine the effectiveness and long-term effect of this exercise modality in overweight and obese patients and to guide future physical activity prescriptions, particularly in terms of exercise modality, intensity and duration. Finally, the combination of ECC and nutritional anabolic compounds as an appropriate source of dietary proteins could be examined in future multimodal approaches for chronic diseases that limit mobility.

## Author contributions

VJ wrote the manuscript with the help of DT, HP, and RR. RR and MD supervised the project. All authors contributed to manuscript revision, read and approved the submitted version.

### Conflict of interest statement

The authors declare that the research was conducted in the absence of any commercial or financial relationships that could be construed as a potential conflict of interest.
